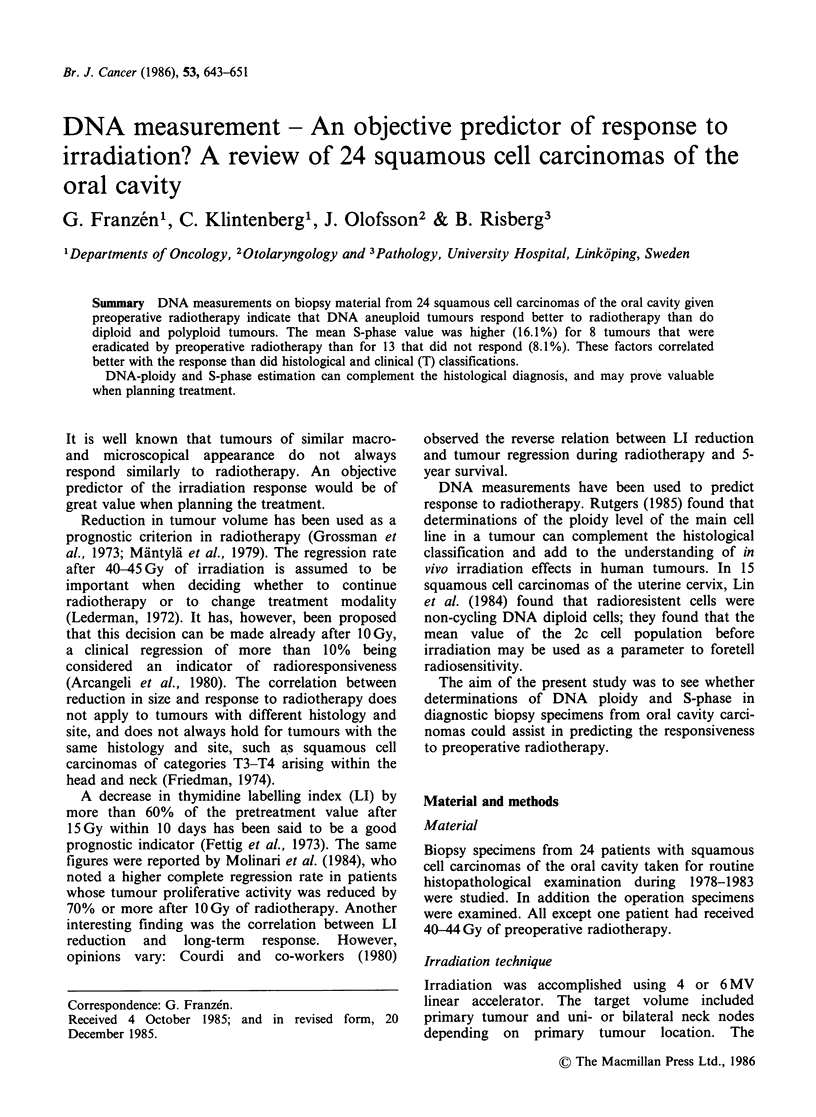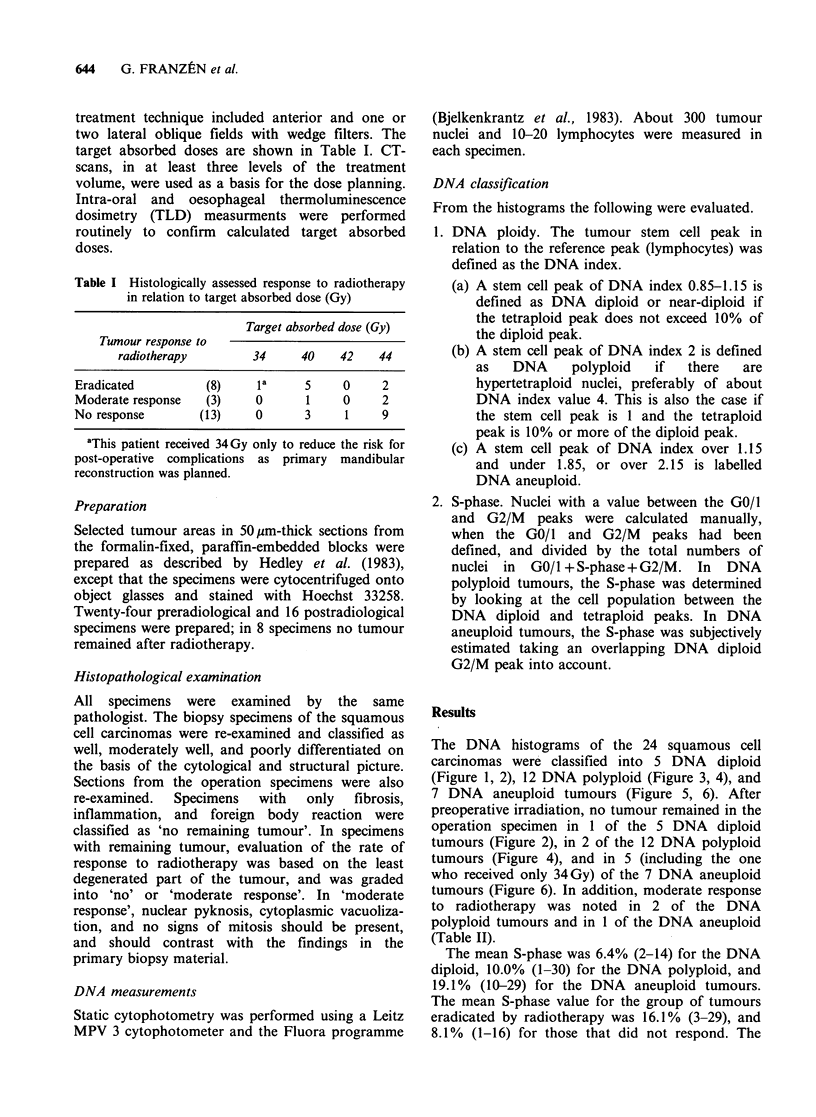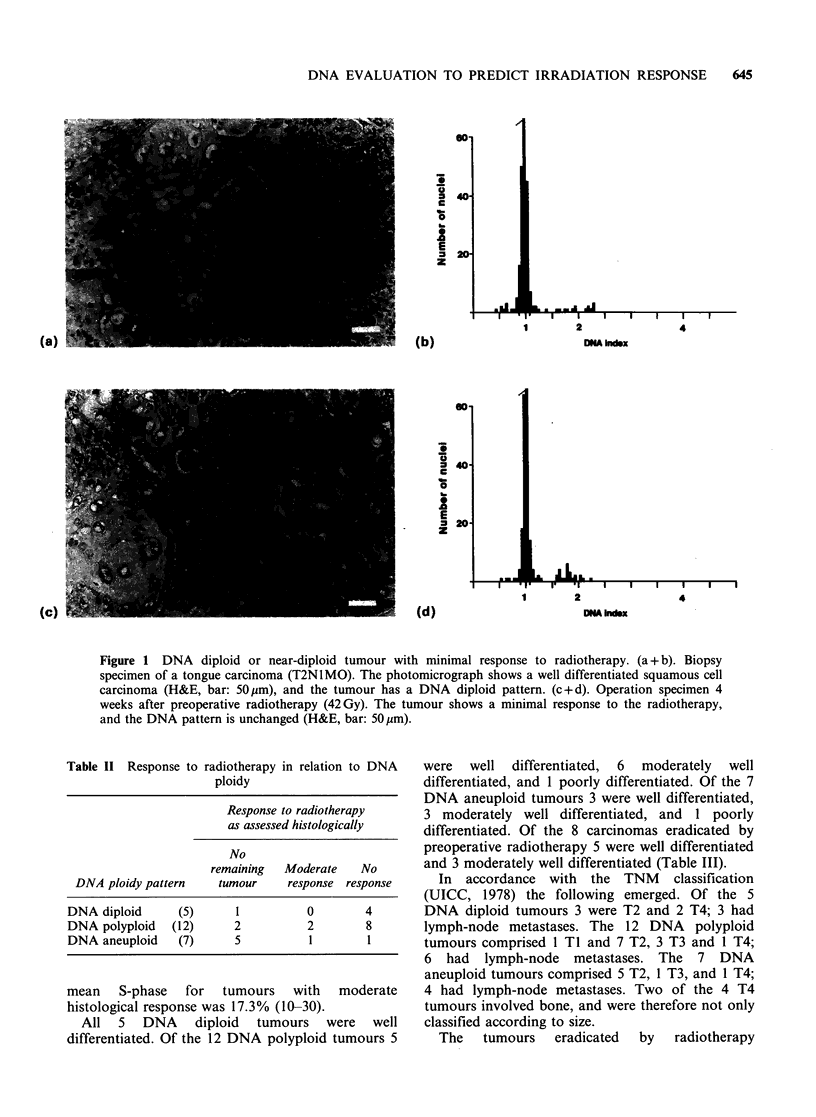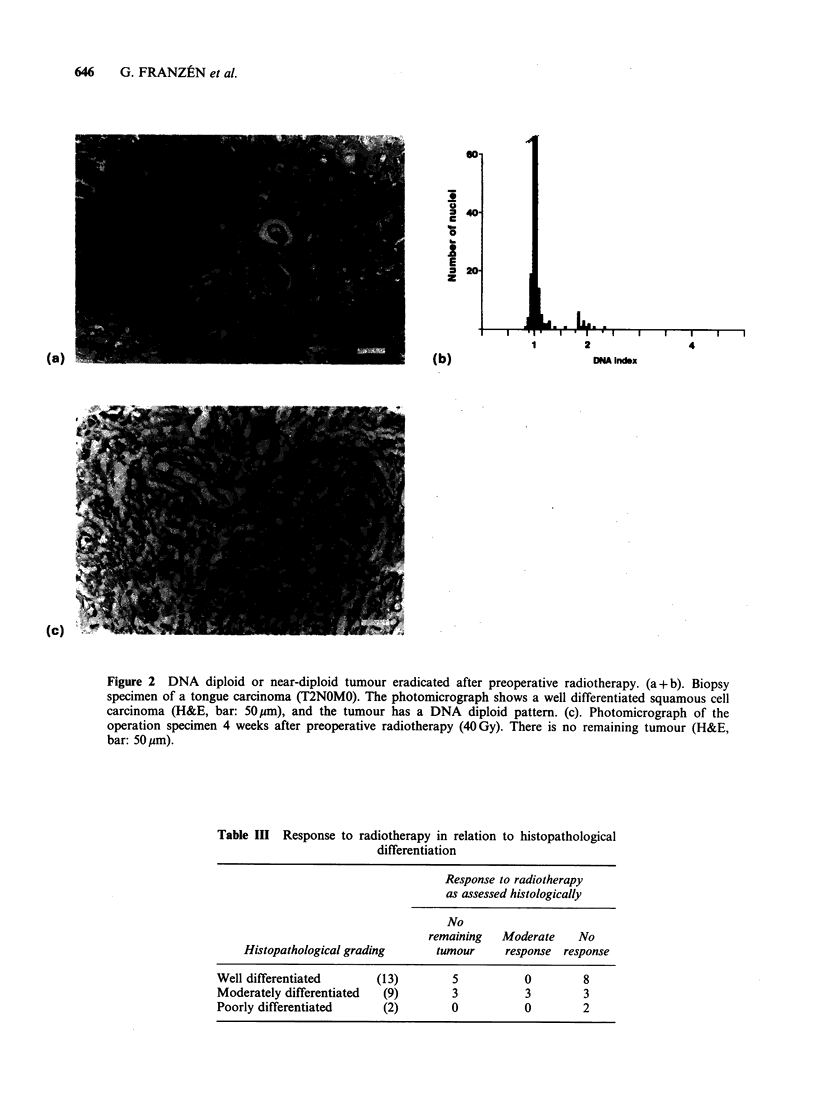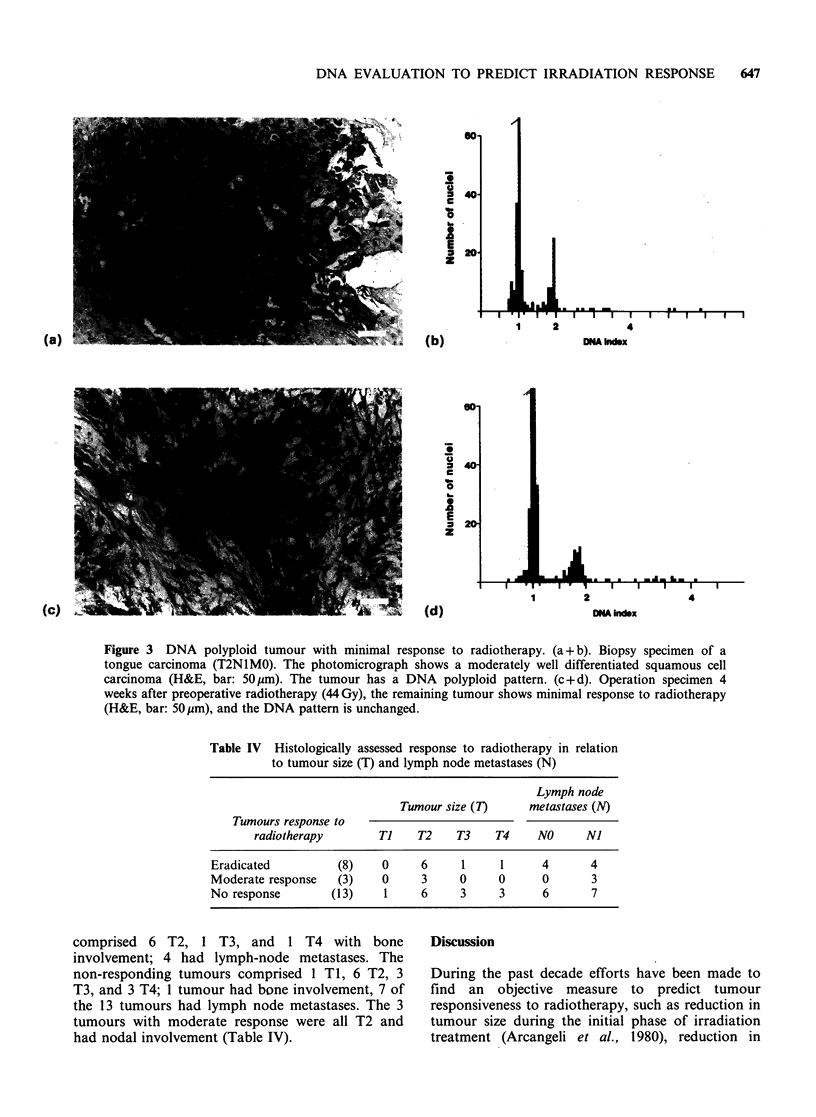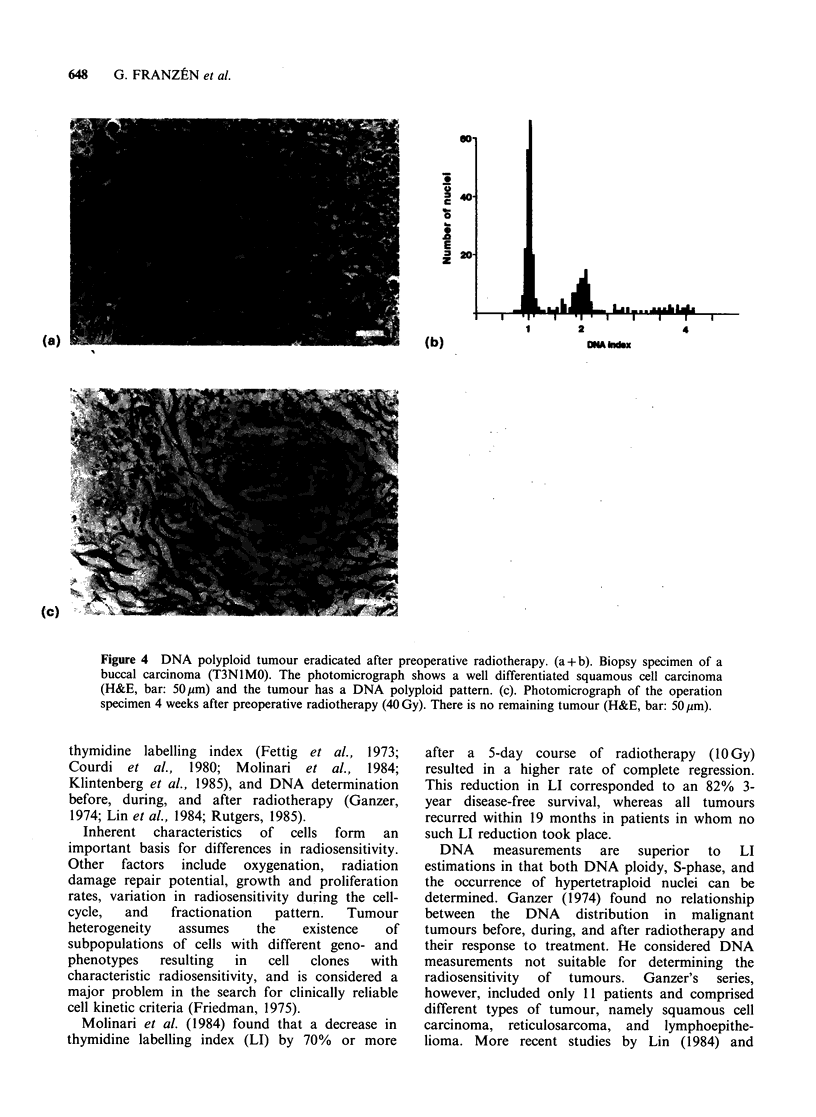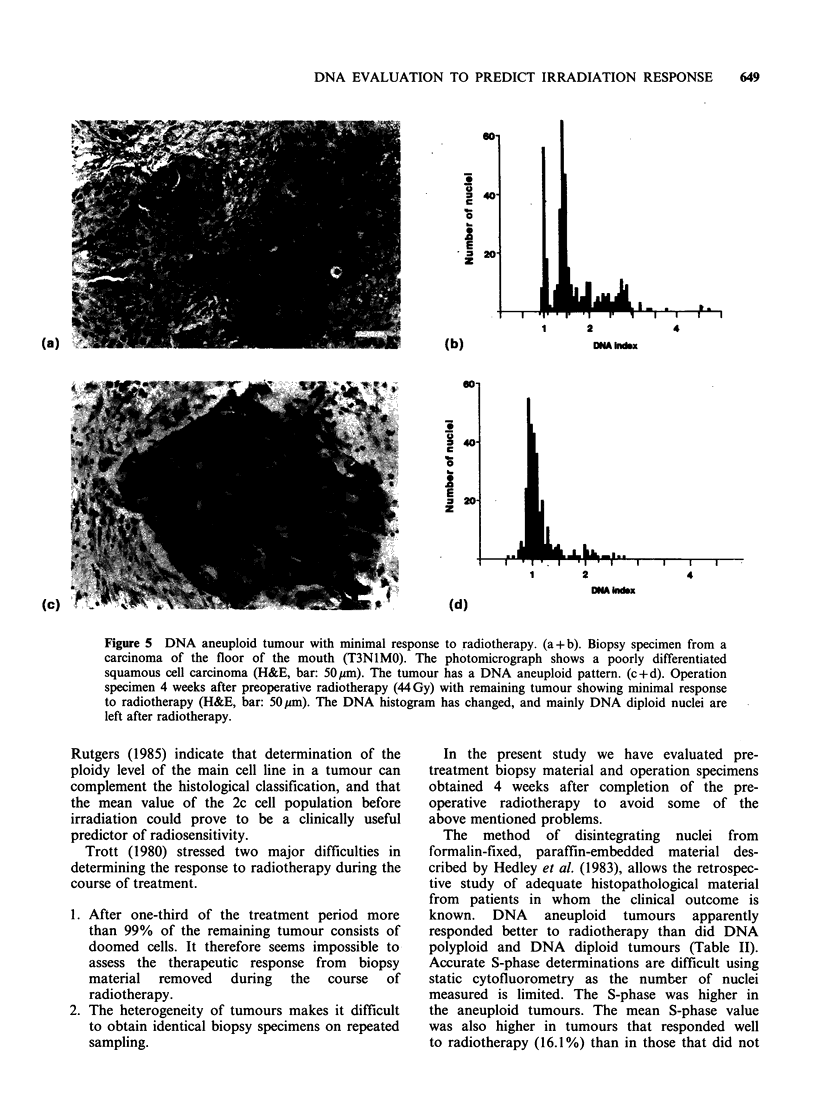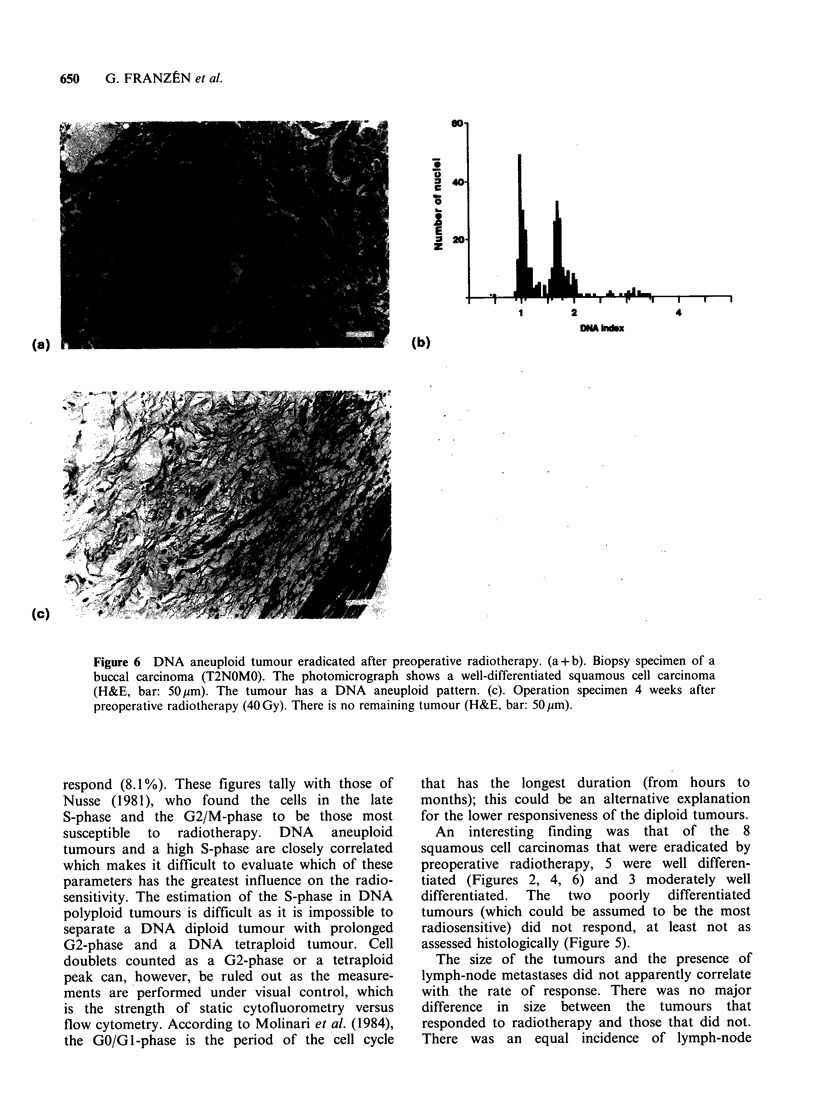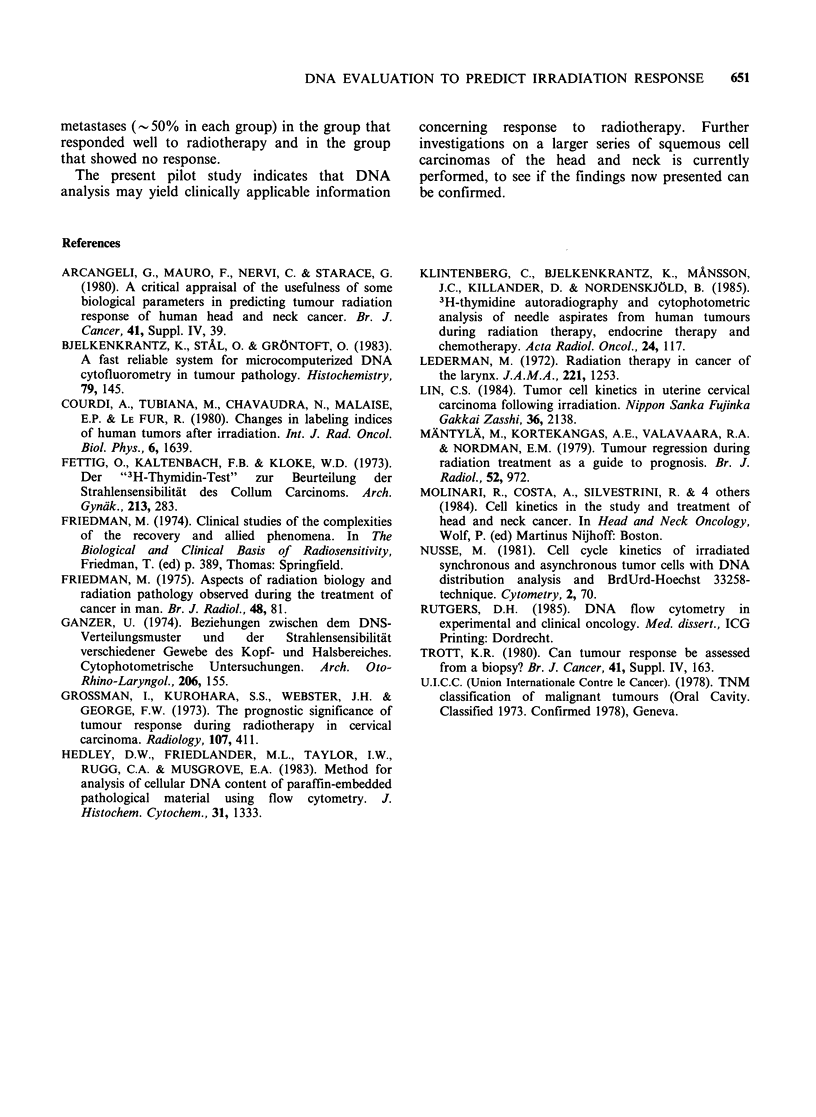# DNA measurement--an objective predictor of response to irradiation? A review of 24 squamous cell carcinomas of the oral cavity.

**DOI:** 10.1038/bjc.1986.108

**Published:** 1986-05

**Authors:** G. Franzén, C. Klintenberg, J. Olofsson, B. Risberg

## Abstract

**Images:**


*Br. J. Cancer* (1986), **53**, 643–651

### DNA measurement – An objective predictor of response to irradiation? A review of 24 squamous cell carcinomas of the oral cavity

G. Franzén[1], C. Klintenberg[1], J. Olofsson[2] & B. Risberg[3]

[1]*Departments of Oncology,* [2]*Otolaryngology and* [3]*Pathology, University Hospital, Linköping, Sweden*

**Summary** DNA measurements on biopsy material from 24 squamous cell carcinomas of the oral cavity given preoperative radiotherapy indicate that DNA aneuploid tumours respond better to radiotherapy than do diploid and polyploid tumours. The mean S-phase value was higher (16.1%) for 8 tumours that were eradicated by preoperative radiotherapy than for 13 that did not respond (8.1%). These factors correlated better with the response than did histological and clinical (T) classifications.

DNA-ploidy and S-phase estimation can complement the histological diagnosis, and may prove valuable when planning treatment.

It is well known that tumours of similar macro- and microscopical appearance do not always respond similarly to radiotherapy. An objective predictor of the irradiation response would be of great value when planning the treatment.

Reduction in tumour volume has been used as a prognostic criterion in radiotherapy (Grossman *et al.*, 1973; Mäntylä *et al.*, 1979). The regression rate after 40–45 Gy of irradiation is assumed to be important when deciding whether to continue radiotherapy or to change treatment modality (Lederman, 1972). It has, however, been proposed that this decision can be made already after 10 Gy, a clinical regression of more than 10% being considered an indicator of radioresponsiveness (Arcangeli *et al.*, 1980). The correlation between reduction in size and response to radiotherapy does not apply to tumours with different histology and site, and does not always hold for tumours with the same histology and site, such as squamous cell carcinomas of categories T3–T4 arising within the head and neck (Friedman, 1974).

A decrease in thymidine labelling index (LI) by more than 60% of the pretreatment value after 15 Gy within 10 days has been said to be a good prognostic indicator (Fettig *et al.*, 1973). The same figures were reported by Molinari *et al.* (1984), who noted a higher complete regression rate in patients whose tumour proliferative activity was reduced by 70% or more after 10 Gy of radiotherapy. Another interesting finding was the correlation between LI reduction and long-term response. However, opinions vary: Courdi and co-workers (1980) observed the reverse relation between LI reduction and tumour regression during radiotherapy and 5-year survival.

DNA measurements have been used to predict response to radiotherapy. Rutgers (1985) found that determinations of the ploidy level of the main cell line in a tumour can complement the histological classification and add to the understanding of *in vivo* irradiation effects in human tumours. In 15 squamous cell carcinomas of the uterine cervix, Lin *et al.* (1984) found that radioresistent cells were non-cycling DNA diploid cells; they found that the mean value of the 2c cell population before irradiation may be used as a parameter to foretell radiosensitivity.

The aim of the present study was to see whether determinations of DNA ploidy and S-phase in diagnostic biopsy specimens from oral cavity carcinomas could assist in predicting the responsiveness to preoperative radiotherapy.

### Material and methods

### *Material*

Biopsy specimens from 24 patients with squamous cell carcinomas of the oral cavity taken for routine histopathological examination during 1978–1983 were studied. In addition the operation specimens were examined. All except one patient had received 40–44 Gy of preoperative radiotherapy.

### *Irradiation technique*

Irradiation was accomplished using 4 or 6 MV linear accelerator. The target volume included primary tumour and uni- or bilateral neck nodes depending on primary tumour location. The

Correspondence: G. Franzén.
Received 4 October 1985; and in revised form, 20 December 1985.

© The Macmillan Press Ltd., 1986

treatment technique included anterior and one or two lateral oblique fields with wedge filters. The target absorbed doses are shown in Table I. CT-scans, in at least three levels of the treatment volume, were used as a basis for the dose planning. Intra-oral and oesophageal thermoluminescence dosimetry (TLD) measurments were performed routinely to confirm calculated target absorbed doses.

**Table I** Histologically assessed response to radiotherapy in relation to target absorbed dose (Gy)

| Tumour response to radiotherapy | | Target absorbed dose (Gy) | | | |
|---|---|---|---|---|---|
| | | *34* | *40* | *42* | *44* |
| Eradicated | (8) | 1[a] | 5 | 0 | 2 |
| Moderate response | (3) | 0 | 1 | 0 | 2 |
| No response | (13) | 0 | 3 | 1 | 9 |

[a]This patient received 34 Gy only to reduce the risk for post-operative complications as primary mandibular reconstruction was planned.

*Preparation*

Selected tumour areas in 50 $\mu$m-thick sections from the formalin-fixed, paraffin-embedded blocks were prepared as described by Hedley *et al.* (1983), except that the specimens were cytocentrifuged onto object glasses and stained with Hoechst 33258. Twenty-four preradiological and 16 postradiological specimens were prepared; in 8 specimens no tumour remained after radiotherapy.

*Histopathological examination*

All specimens were examined by the same pathologist. The biopsy specimens of the squamous cell carcinomas were re-examined and classified as well, moderately well, and poorly differentiated on the basis of the cytological and structural picture. Sections from the operation specimens were also re-examined. Specimens with only fibrosis, inflammation, and foreign body reaction were classified as 'no remaining tumour'. In specimens with remaining tumour, evaluation of the rate of response to radiotherapy was based on the least degenerated part of the tumour, and was graded into 'no' or 'moderate response'. In 'moderate response', nuclear pyknosis, cytoplasmic vacuolization, and no signs of mitosis should be present, and should contrast with the findings in the primary biopsy material.

*DNA measurements*

Static cytophotometry was performed using a Leitz MPV 3 cytophotometer and the Fluora programme (Bjelkenkrantz *et al.,* 1983). About 300 tumour nuclei and 10–20 lymphocytes were measured in each specimen.

*DNA classification*

From the histograms the following were evaluated.

1. DNA ploidy. The tumour stem cell peak in relation to the reference peak (lymphocytes) was defined as the DNA index.
   (a) A stem cell peak of DNA index 0.85–1.15 is defined as DNA diploid or near-diploid if the tetraploid peak does not exceed 10% of the diploid peak.
   (b) A stem cell peak of DNA index 2 is defined as DNA polyploid if there are hypertetraploid nuclei, preferably of about DNA index value 4. This is also the case if the stem cell peak is 1 and the tetraploid peak is 10% or more of the diploid peak.
   (c) A stem cell peak of DNA index over 1.15 and under 1.85, or over 2.15 is labelled DNA aneuploid.
2. S-phase. Nuclei with a value between the G0/1 and G2/M peaks were calculated manually, when the G0/1 and G2/M peaks had been defined, and divided by the total numbers of nuclei in G0/1 + S-phase + G2/M. In DNA polyploid tumours, the S-phase was determined by looking at the cell population between the DNA diploid and tetraploid peaks. In DNA aneuploid tumours, the S-phase was subjectively estimated taking an overlapping DNA diploid G2/M peak into account.

### Results

The DNA histograms of the 24 squamous cell carcinomas were classified into 5 DNA diploid (Figure 1, 2), 12 DNA polyploid (Figure 3, 4), and 7 DNA aneuploid tumours (Figure 5, 6). After preoperative irradiation, no tumour remained in the operation specimen in 1 of the 5 DNA diploid tumours (Figure 2), in 2 of the 12 DNA polyploid tumours (Figure 4), and in 5 (including the one who received only 34 Gy) of the 7 DNA aneuploid tumours (Figure 6). In addition, moderate response to radiotherapy was noted in 2 of the DNA polyploid tumours and in 1 of the DNA aneuploid (Table II).

The mean S-phase was 6.4% (2–14) for the DNA diploid, 10.0% (1–30) for the DNA polyploid, and 19.1% (10–29) for the DNA aneuploid tumours. The mean S-phase value for the group of tumours eradicated by radiotherapy was 16.1% (3–29), and 8.1% (1–16) for those that did not respond. The

**Figure 1** DNA diploid or near-diploid tumour with minimal response to radiotherapy. (a+b). Biopsy specimen of a tongue carcinoma (T2N1MO). The photomicrograph shows a well differentiated squamous cell carcinoma (H&E, bar: 50 $\mu$m), and the tumour has a DNA diploid pattern. (c+d). Operation specimen 4 weeks after preoperative radiotherapy (42 Gy). The tumour shows a minimal response to the radiotherapy, and the DNA pattern is unchanged (H&E, bar: 50 $\mu$m).

**Table II** Response to radiotherapy in relation to DNA ploidy

| | *Response to radiotherapy as assessed histologically* | | |
|---|---|---|---|
| *DNA ploidy pattern* | *No remaining tumour* | *Moderate response* | *No response* |
| DNA diploid (5) | 1 | 0 | 4 |
| DNA polyploid (12) | 2 | 2 | 8 |
| DNA aneuploid (7) | 5 | 1 | 1 |

mean S-phase for tumours with moderate histological response was 17.3% (10–30).

All 5 DNA diploid tumours were well differentiated. Of the 12 DNA polyploid tumours 5 were well differentiated, 6 moderately well differentiated, and 1 poorly differentiated. Of the 7 DNA aneuploid tumours 3 were well differentiated, 3 moderately well differentiated, and 1 poorly differentiated. Of the 8 carcinomas eradicated by preoperative radiotherapy 5 were well differentiated and 3 moderately well differentiated (Table III).

In accordance with the TNM classification (UICC, 1978) the following emerged. Of the 5 DNA diploid tumours 3 were T2 and 2 T4; 3 had lymph-node metastases. The 12 DNA polyploid tumours comprised 1 T1 and 7 T2, 3 T3 and 1 T4; 6 had lymph-node metastases. The 7 DNA aneuploid tumours comprised 5 T2, 1 T3, and 1 T4; 4 had lymph-node metastases. Two of the 4 T4 tumours involved bone, and were therefore not only classified according to size.

The tumours eradicated by radiotherapy

**Figure 2** DNA diploid or near-diploid tumour eradicated after preoperative radiotherapy. (a + b). Biopsy specimen of a tongue carcinoma (T2N0M0). The photomicrograph shows a well differentiated squamous cell carcinoma (H&E, bar: 50 $\mu$m), and the tumour has a DNA diploid pattern. (c). Photomicrograph of the operation specimen 4 weeks after preoperative radiotherapy (40 Gy). There is no remaining tumour (H&E, bar: 50 $\mu$m).

**Table III** Response to radiotherapy in relation to histopathological differentiation

| | | *Response to radiotherapy as assessed histologically* | | |
|---|---|---|---|---|
| *Histopathological grading* | | *No remaining tumour* | *Moderate response* | *No response* |
| Well differentiated | (13) | 5 | 0 | 8 |
| Moderately differentiated | (9) | 3 | 3 | 3 |
| Poorly differentiated | (2) | 0 | 0 | 2 |

**Figure 3** DNA polyploid tumour with minimal response to radiotherapy. (a+b). Biopsy specimen of a tongue carcinoma (T2N1M0). The photomicrograph shows a moderately well differentiated squamous cell carcinoma (H&E, bar: 50 μm). The tumour has a DNA polyploid pattern. (c+d). Operation specimen 4 weeks after preoperative radiotherapy (44 Gy), the remaining tumour shows minimal response to radiotherapy (H&E, bar: 50 μm), and the DNA pattern is unchanged.

**Table IV** Histologically assessed response to radiotherapy in relation to tumour size (T) and lymph node metastases (N)

| *Tumours response to radiotherapy* | | *Tumour size (T)* | | | | *Lymph node metastases (N)* | |
|---|---|---|---|---|---|---|---|
| | | *T1* | *T2* | *T3* | *T4* | *N0* | *N1* |
| Eradicated | (8) | 0 | 6 | 1 | 1 | 4 | 4 |
| Moderate response | (3) | 0 | 3 | 0 | 0 | 0 | 3 |
| No response | (13) | 1 | 6 | 3 | 3 | 6 | 7 |

comprised 6 T2, 1 T3, and 1 T4 with bone involvement; 4 had lymph-node metastases. The non-responding tumours comprised 1 T1, 6 T2, 3 T3, and 3 T4; 1 tumour had bone involvement, 7 of the 13 tumours had lymph node metastases. The 3 tumours with moderate response were all T2 and had nodal involvement (Table IV).

### Discussion

During the past decade efforts have been made to find an objective measure to predict tumour responsiveness to radiotherapy, such as reduction in tumour size during the initial phase of irradiation treatment (Arcangeli *et al.*, 1980), reduction in

**Figure 4** DNA polyploid tumour eradicated after preoperative radiotherapy. (a + b). Biopsy specimen of a buccal carcinoma (T3N1M0). The photomicrograph shows a well differentiated squamous cell carcinoma (H&E, bar: 50 μm) and the tumour has a DNA polyploid pattern. (c). Photomicrograph of the operation specimen 4 weeks after preoperative radiotherapy (40 Gy). There is no remaining tumour (H&E, bar: 50 μm).

thymidine labelling index (Fettig *et al.,* 1973; Courdi *et al.,* 1980; Molinari *et al.,* 1984; Klintenberg *et al.,* 1985), and DNA determination before, during, and after radiotherapy (Ganzer, 1974; Lin *et al.,* 1984; Rutgers, 1985).

Inherent characteristics of cells form an important basis for differences in radiosensitivity. Other factors include oxygenation, radiation damage repair potential, growth and proliferation rates, variation in radiosensitivity during the cell-cycle, and fractionation pattern. Tumour heterogeneity assumes the existence of subpopulations of cells with different geno- and phenotypes resulting in cell clones with characteristic radiosensitivity, and is considered a major problem in the search for clinically reliable cell kinetic criteria (Friedman, 1975).

Molinari *et al.* (1984) found that a decrease in thymidine labelling index (LI) by 70% or more after a 5-day course of radiotherapy (10 Gy) resulted in a higher rate of complete regression. This reduction in LI corresponded to an 82% 3-year disease-free survival, whereas all tumours recurred within 19 months in patients in whom no such LI reduction took place.

DNA measurements are superior to LI estimations in that both DNA ploidy, S-phase, and the occurrence of hypertetraploid nuclei can be determined. Ganzer (1974) found no relationship between the DNA distribution in malignant tumours before, during, and after radiotherapy and their response to treatment. He considered DNA measurements not suitable for determining the radiosensitivity of tumours. Ganzer's series, however, included only 11 patients and comprised different types of tumour, namely squamous cell carcinoma, reticulosarcoma, and lymphoepithelioma. More recent studies by Lin (1984) and

**Figure 5** DNA aneuploid tumour with minimal response to radiotherapy. (a+b). Biopsy specimen from a carcinoma of the floor of the mouth (T3N1M0). The photomicrograph shows a poorly differentiated squamous cell carcinoma (H&E, bar: 50 μm). The tumour has a DNA aneuploid pattern. (c+d). Operation specimen 4 weeks after preoperative radiotherapy (44 Gy) with remaining tumour showing minimal response to radiotherapy (H&E, bar: 50 μm). The DNA histogram has changed, and mainly DNA diploid nuclei are left after radiotherapy.

Rutgers (1985) indicate that determination of the ploidy level of the main cell line in a tumour can complement the histological classification, and that the mean value of the 2c cell population before irradiation could prove to be a clinically useful predictor of radiosensitivity.

Trott (1980) stressed two major difficulties in determining the response to radiotherapy during the course of treatment.

1. After one-third of the treatment period more than 99% of the remaining tumour consists of doomed cells. It therefore seems impossible to assess the therapeutic response from biopsy material removed during the course of radiotherapy.
2. The heterogeneity of tumours makes it difficult to obtain identical biopsy specimens on repeated sampling.

In the present study we have evaluated pre-treatment biopsy material and operation specimens obtained 4 weeks after completion of the pre-operative radiotherapy to avoid some of the above mentioned problems.

The method of disintegrating nuclei from formalin-fixed, paraffin-embedded material described by Hedley *et al.* (1983), allows the retrospective study of adequate histopathological material from patients in whom the clinical outcome is known. DNA aneuploid tumours apparently responded better to radiotherapy than did DNA polyploid and DNA diploid tumours (Table II). Accurate S-phase determinations are difficult using static cytofluorometry as the number of nuclei measured is limited. The S-phase was higher in the aneuploid tumours. The mean S-phase value was also higher in tumours that responded well to radiotherapy (16.1%) than in those that did not

**Figure 6** DNA aneuploid tumour eradicated after preoperative radiotherapy. (a + b). Biopsy specimen of a buccal carcinoma (T2N0M0). The photomicrograph shows a well-differentiated squamous cell carcinoma (H&E, bar: 50 $\mu$m). The tumour has a DNA aneuploid pattern. (c). Operation specimen 4 weeks after preoperative radiotherapy (40 Gy). There is no remaining tumour (H&E, bar: 50 $\mu$m).

respond (8.1%). These figures tally with those of Nusse (1981), who found the cells in the late S-phase and the G2/M-phase to be those most susceptible to radiotherapy. DNA aneuploid tumours and a high S-phase are closely correlated which makes it difficult to evaluate which of these parameters has the greatest influence on the radiosensitivity. The estimation of the S-phase in DNA polyploid tumours is difficult as it is impossible to separate a DNA diploid tumour with prolonged G2-phase and a DNA tetraploid tumour. Cell doublets counted as a G2-phase or a tetraploid peak can, however, be ruled out as the measurements are performed under visual control, which is the strength of static cytofluorometry versus flow cytometry. According to Molinari *et al.* (1984), the G0/G1-phase is the period of the cell cycle that has the longest duration (from hours to months); this could be an alternative explanation for the lower responsiveness of the diploid tumours.

An interesting finding was that of the 8 squamous cell carcinomas that were eradicated by preoperative radiotherapy, 5 were well differentiated (Figures 2, 4, 6) and 3 moderately well differentiated. The two poorly differentiated tumours (which could be assumed to be the most radiosensitive) did not respond, at least not as assessed histologically (Figure 5).

The size of the tumours and the presence of lymph-node metastases did not apparently correlate with the rate of response. There was no major difference in size between the tumours that responded to radiotherapy and those that did not. There was an equal incidence of lymph-node metastases ($\sim$50% in each group) in the group that responded well to radiotherapy and in the group that showed no response.

The present pilot study indicates that DNA analysis may yield clinically applicable information concerning response to radiotherapy. Further investigations on a larger series of squemous cell carcinomas of the head and neck is currently performed, to see if the findings now presented can be confirmed.

### References

ARCANGELI, G., MAURO, F., NERVI, C. & STARACE, G. (1980). A critical appraisal of the usefulness of some biological parameters in predicting tumour radiation response of human head and neck cancer. *Br. J. Cancer*, **41**, Suppl. IV, 39.

BJELKENKRANTZ, K., STÅL, O. & GRÖNTOFT, O. (1983). A fast reliable system for microcomputerized DNA cytofluorometry in tumour pathology. *Histochemistry*, **79**, 145.

COURDI, A., TUBIANA, M., CHAVAUDRA, N., MALAISE, E.P. & LE FUR, R. (1980). Changes in labeling indices of human tumors after irradiation. *Int. J. Rad. Oncol. Biol. Phys.*, **6**, 1639.

FETTIG, O., KALTENBACH, F.B. & KLOKE, W.D. (1973). Der "$^3$H-Thymidin-Test" zur Beurteilung der Strahlensensibilität des Collum Carcinoms. *Arch. Gynäk.*, **213**, 283.

FRIEDMAN, M. (1974). Clinical studies of the complexities of the recovery and allied phenomena. In *The Biological and Clinical Basis of Radiosensitivity*, Friedman, T. (ed) p. 389, Thomas: Springfield.

FRIEDMAN, M. (1975). Aspects of radiation biology and radiation pathology observed during the treatment of cancer in man. *Br. J. Radiol.*, **48**, 81.

GANZER, U. (1974). Beziehungen zwischen dem DNS-Verteilungsmuster und der Strahlensensibilität verschiedener Gewebe des Kopf- und Halsbereiches. Cytophotometrische Untersuchungen. *Arch. Oto-Rhino-Laryngol.*, **206**, 155.

GROSSMAN, I., KUROHARA, S.S., WEBSTER, J.H. & GEORGE, F.W. (1973). The prognostic significance of tumour response during radiotherapy in cervical carcinoma. *Radiology*, **107**, 411.

HEDLEY, D.W., FRIEDLANDER, M.L., TAYLOR, I.W., RUGG, C.A. & MUSGROVE, E.A. (1983). Method for analysis of cellular DNA content of paraffin-embedded pathological material using flow cytometry. *J. Histochem. Cytochem.*, **31**, 1333.

KLINTENBERG, C., BJELKENKRANTZ, K., MÅNSSON, J.C., KILLANDER, D. & NORDENSKJÖLD, B. (1985). $^3$H-thymidine autoradiography and cytophotometric analysis of needle aspirates from human tumours during radiation therapy, endocrine therapy and chemotherapy. *Acta Radiol. Oncol.*, **24**, 117.

LEDERMAN, M. (1972). Radiation therapy in cancer of the larynx. *J.A.M.A.*, **221**, 1253.

LIN, C.S. (1984). Tumor cell kinetics in uterine cervical carcinoma following irradiation. *Nippon Sanka Fujinka Gakkai Zasshi*, **36**, 2138.

MÄNTYLÄ, M., KORTEKANGAS, A.E., VALAVAARA, R.A. & NORDMAN, E.M. (1979). Tumour regression during radiation treatment as a guide to prognosis. *Br. J. Radiol.*, **52**, 972.

MOLINARI, R., COSTA, A., SILVESTRINI, R. & 4 others (1984). Cell kinetics in the study and treatment of head and neck cancer. In *Head and Neck Oncology*, Wolf, P. (ed) Martinus Nijhoff: Boston.

NUSSE, M. (1981). Cell cycle kinetics of irradiated synchronous and asynchronous tumor cells with DNA distribution analysis and BrdUrd-Hoechst 33258-technique. *Cytometry*, **2**, 70.

RUTGERS, D.H. (1985). DNA flow cytometry in experimental and clinical oncology. *Med. dissert.*, ICG Printing: Dordrecht.

TROTT, K.R. (1980). Can tumour response be assessed from a biopsy? *Br. J. Cancer*, **41**, Suppl. IV, 163.

U.I.C.C. (Union Internationale Contre le Cancer). (1978). TNM classification of malignant tumours (Oral Cavity. Classified 1973. Confirmed 1978), Geneva.